# Consensus on innovations and future directions of community first responder schemes in United Kingdom: a national nominal group technique study

**DOI:** 10.1186/s13049-024-01254-6

**Published:** 2024-09-30

**Authors:** Gupteswar Patel, Vanessa Botan, Viet-Hai Phung, Ian Trueman, Mehrshad Parvin Hosseini, Murray D. Smith, Roderick Ørner, Julie Pattinson, Zahid Asghar, Elise Rowan, Robert Spaight, Craig Mortimer, Amanda Brewster, Pauline Mountain, Joshua Miller, Martina Brown, Aloysius Niroshan Siriwardena

**Affiliations:** 1 Community and Health Research Unit, School of Health and Care Sciences, University of Lincoln, Lincoln, UK; 2 Aberystwyth Business School, Aberystwyth University, Ceredigion, UK; 3 East Midlands Ambulance Service NHS Trust, Nottingham, UK; 4 South East Coast Ambulance Service NHS Foundation Trust, Sussex, UK; 5 West Midlands Ambulance Service NHS Trust, Birmingham, UK; 6 South Central Ambulance Service NHS Foundation Trust, Bicester, UK

**Keywords:** Community First Responder, Innovations, Nominal group technique, United Kingdom

## Abstract

**Aim:**

We aimed to achieve consensus among NHS and community stakeholders to identify and prioritise innovations in Community First Responder (CFR) schemes.

**Methods:**

We conducted a mixed-methods study, adopting a modified nominal group technique with participants from ambulance services, CFR schemes and community stakeholders. The 1-day consensus workshop consisted of four sessions: introduction of innovations derived from primary research; round-robin discussions to generate new ideas; discussion and ranking of innovations; feedback of ranking, re-ranking and concluding statements. Innovations were ranked on a 5-point Likert scale and descriptive statistics of median and interquartile range calculated. Discussions were recorded, transcribed, and analysed thematically.

**Results:**

The innovations found were classified into two categories: process innovations and technological innovations. The process innovations included six types of innovations: roles, governance, training, policies and protocols, recruitment, and awareness. The technological innovations included three aspects: information and communication; transport; and health technology. The descriptive statistics revealed that innovations such as counselling and support for CFRs (median: 5 IQR 5,5), peer support [5 (4,5)], and enhanced communication with control room [5 (4,5)] were essential priorities. Contrastingly, innovations such as the provision of dual CFR crew [1.5 (1,3)], CFR responsibilities in patient transport to hospital [1 (1,2)], and CFR access to emergency blue light [1 (1,1.5)] were deemed non-priorities.

**Conclusions:**

This article established consensus on innovations in the CFR schemes and their ranking for improving the provision of care delivered by CFRs in communities. The consensus-building process also informed policy- and decision-makers on the potential future change agenda for CFR schemes.

**Supplementary Information:**

The online version contains supplementary material available at 10.1186/s13049-024-01254-6.

## Introduction

Community First Responder (CFR) schemes in the UK provide emergency and prehospital care, particularly in remote and rural areas, closely linked with Emergency Medical Services (EMS) [1, 2]. In the UK, ambulance and EMS mobilise CFR volunteers to respond to emergencies within their locality [3, 4]. Ambulance services in England recruit CFRs from a pool of interested lay volunteers, members of the public who through training in Basic Life Support (BLS) have the necessary skills and equipment to respond to emergencies. Although CFR schemes have been active in the UK since 1999, this remains an underexplored area in healthcare research. Previous research has largely focused on the motivation to become a CFR [4–7] operational strategies and challenges [5, 8], improving access to emergency care [9], the role of CFRs in rural healthcare [10], and CFR practice [11]. Relatively little is known about the aspects of innovations of CFRs and CFR schemes that have been implemented by different ambulance services and trusts. To address this lack of evidence on CFR roles and innovations, we undertook a CFR study across six ambulance service regions of England.

In the UK, around 2,500 CFR schemes exist, run either by independent charities or ambulance trusts. Previous studies have noted the absence of a universal or national standard for CFR training, support, scope of practice, quality standards and strategies for using CFRs varies between services [8, 12]. Hence, it is important to recognise the innovations in CFR operations and understand their importance from the perspectives of CFR operational stakeholders for the future roles of CFRs in health care provision.

Using a Nominal Group Technique (NGT), we aimed to document innovations in CFR schemes and use consensus methods involving key stakeholders to prioritise innovations for future rural healthcare. There is a particular focus on rural healthcare as they are more likely to experience geographical isolation, which can lead to slower ambulance response times. CFRs located in rural areas are often able to arrive on-scene faster than ambulance crews, and contribute more significantly to prehospital care delivery, in particular, response times, in rural areas [10].

The consensus approach is a process and a collection of scientific methods that enable stakeholders with relevant expertise to agree priority areas in policy or decision-making [13]. The consensus approach relies on generating high-quality evidence for healthcare. NGT is one of the most common consensus approaches recognised by the National Institute of Clinical Excellence in the UK [14].

The consensus study, which is the focus of this paper, is part of a comprehensive CFR work programme, ‘Community First Responders’ role in the current and future rural healthcare workforce (https://www.fundingawards.nihr.ac.uk/award/NIHR127920: 2020–2022). This overall work programme sought to establish evidence of what CFRs did, how they did this, what outcomes were achieved and what is planned for their future development. The first part of the programme was a quantitative analysis of routine data from six English ambulance services database that identified the rate and type of ambulance calls attended by CFRs [10]. The quantitative study was followed by an econometric analysis of primary and secondary data gathered from the ambulance services to identify costs of the CFR schemes provision, sources of funding and evaluate outcomes of CFR schemes. Preceding the consensus study was a qualitative exploration of experiences, perceptions, challenges, and opportunities of different stakeholders involved in CFR scheme implementation, including CFRs, CFR leads, ambulance staff, and commissioners [8, 11].

This paper relates to the last segment of the CFR programme, which aimed to achieve consensus among CFR scheme stakeholders as well as public representatives to identify and prioritise innovations in CFR schemes. This will identify and inform future innovations and implementation plan for improving CFR schemes.

## Methods

### Design

Methodologically, this was a mixed-methods study that sought to combine the advantages and minimise the disadvantages of quantitative and qualitative research, while establishing consensus on CFR innovations. Quantitative research holds that there is one objective, value-free, measurable reality, detached from its context [15, 16]. It is explanatory because, as Biesta explained, it aims “to identify causes, factors or correlations and through this, generate knowledge that can be used to influence the course of future events” [17]. Unlike quantitative research, qualitative research allows for exploration and understanding of multiple subjective realities. While quantitative research answers the where, what, when, and who, qualitative research explores the how and why [18–20]. Therefore, in this consensus study, we adopted qualitative methods to explore and identify innovations for future CFR schemes and quantitative methods to conduct surveys for voting on the identified innovations, and their prioritisation using descriptive statistics.

The NGT is “a structured, well-established, multistep, facilitated, group meeting technique used to generate and prioritise responses to a specific question by a group of people who have expert insight into a particular area of interest.” [21]. Ours is a modified NGT because it includes a virtual element, alongside the face-to-face meeting, due to travel constraints and risk to some participants of infection (COVID-19) [22, 23]. Our methods are consistent with the previous research that recognises the value of online meetings for consensus building exercises [24].

Ethical approval was received from the NHS Research Ethics Committee (IRAS project ID—277205, registration reference: NCT04279262).

### The consensus workshop

NGT, whether conducted face-to-face or virtually produces qualitative (group discussions) and quantitative data (prioritisation through voting) [25]. Researchers facilitate group discussions to enable all participants to contribute to discussions and development of innovations. NGT can be used either as a consensus building process or ranking exercise [26, 27]. In this study, we applied the NGT methods to generate ideas for innovations about how to improve the functionality of CFRs and rank them. Healthcare innovations are generally referred to as ‘new or improved’ health practices, systems, technology, and services in order to improve healthcare delivery [28, 29]. Our study focuses on the latter.

We aimed to achieve a consensus among participants over the innovations relevant to the current and future roles of CFRs. Participant discussions sought to identify and prioritise these innovations. Our modified Nominal Group Technique (mNGT) [13] included four stages: deductive introduction of empirical results and innovations identified from the research; round-robin ideas, where each participant is offered an opportunity to make suggestions, and generation of innovations; clarification and voting on priorities; and final remarks. Each of the three groups enabled participant discussion on priorities. Subsequently, participants voted on these priorities. These processes were organised in a 1-day workshop (see Table 1).

**Table 1:** Characteristics of consensus participant groups

Groups	Designations/roles	Ambulance services OR regions	Gender	Mode of attendance	Voted
Group 1	Regional Blue Light Coordinator and Community Engagement Manager	North West Ambulance Service NHS Trust	Male	In-Person	Yes
	Community Defibrillation officer	Yorkshire Ambulance Service NHS Trust	Male	In-Person	Yes
	PPI member		Female	In-Person	Yes
	Research team			In-Person	No
	Research team			In-Person	No
	Research team			In-Person	No
	Research team			In-Person	No
Group 2	Senior Manager, and Head of Volunteering and Community Services	South West Ambulance Service NHS Foundation Trust	Female	Online	Yes
	Research Paramedic	West Midland Ambulance Service	Male	Online	Yes
	Community Defibrillation officer	Yorkshire Ambulance Service NHS Trust	Male	Online	Yes
	Assistant Chief Ambulance Officer	West Midlands Ambulance Service	Male	Online	Yes
	Community Response Manager	West Midlands Ambulance Service	Male	Online	Yes
	PPI member		Male	Online	Yes
	PPI member		Female	Online	Yes
	Research team			In-Person	No
	Research team			In-Person	No
	Research team			In-Person	No
Group 3	Community Response Officer	Isle of Wight NHS Trust	Male	Online	Yes
	Research Manager	South East Coast Ambulance Service NHS Trust	Male	Online	Yes
	Research Coordinator	Yorkshire Ambulance Service NHS Trust	Female	Online	Yes
	Head of Community Response	East of England Ambulance Service	Female	Online	Yes
	PPI member		Female	Online	Yes
	PPI member		Female	Online	Yes
	PPI member		Male	Online	Yes
	Research team			In-Person	No
	Research team			In-Person	No

Throughout our research and dissemination processes, we communicated with several research leads and decision-makers from various ambulance services. The PPI participants were selected from the PPI group involved in the CFR programme, ensuring representation. Invitations were extended to active PPI members who regularly contributed lay patient and public perspectives during quarterly project meetings. A total of 19 known stakeholders, including representatives from PPI and seven ambulance service institutions in England, who had experiences and in-depth understanding of CFR schemes functions and were able to contribute to the consensus, were invited. One PPI representative and an ambulance service representative were unable to join due to scheduling conflicts and a change in employment, respectively. Additionally, two ambulance service members recommended their colleagues with similar roles, who were subsequently invited and participated with informed consent in the consensus workshop. 17 stakeholders from PPI, and ambulance services participated in the hybrid consensus meeting. The ambulance service stakeholders and PPI representatives had no prior working relationships, with the exception of one PPI member who also volunteered as a CFR. The multidisciplinary representation of the panel was ensured by involving members from CFR representatives, ambulance clinicians and paramedic researchers, commissioners, and PPI representatives. The participants held expertise in functions of CFR, implementation of CFR schemes, understanding of the NHS, and social determinants of EMS. Potential participants were approached by the University of Lincoln research team via emails to confirm their suitability and preference for participation through either MS Teams meeting or in-person at the Lincoln Medical School. A list of participants was finalised based on acquiring their informed consent to participate. Only the PPI participants were financially supported to attend the consensus meeting, and their travel costs were reimbursed.

*Session one* involved a series of three presentations from the research team to introduce the empirical research results. The three presentations focused on: quantitative descriptive analysis of CFR roles and attendance in emergency care; econometric analysis of the CFR and the CFR schemes; and qualitative results of the perceptions and experiences of the different stakeholders involved in the CFR scheme implementation [30]. The qualitative results also included a list of innovations embedded in the CFR schemes and rurality to contextualise what participants were asked to base their ideas on and develop future innovations.

*Session two* built on the discussion from session one and focused on supplementing ideas and innovations for the CFR schemes. The participants were organised into three groups: two groups over MS Teams and one group face-to-face in the consensus workshop venue (see Table 1). All ambulance staff and PPI members were purposively selected to participate in the consensus workshop based on their roles, experiences, and specialist knowledge of the subject under discussion. The two online groups were conducted in two separate rooms and each facilitated by two researchers. At this stage, discussion and generation of ideas, round-robin listing of ideas and innovations occurred concurrently. The facilitators guided the discussion, non-participatorily observed and documented the discussions, and listed the innovations in their respective groups. The group discussions were also recorded with the consent of the participants and transcribed. The salient core categories of innovations discussed in these sessions were noted and included in the list of innovations, which was used for prioritisation through voting using an online survey, hosted by the Jisc platform (https://www.jisc.ac.uk/).

At the end of session two, all the innovations and ideas from empirical results and consensus group discussions were collated and entered into Jisc online survey (https://www.jisc.ac.uk/). A total of 40 innovations were listed for the survey using a Likert scale (1-2-3-4-5), where 1 was the Not a priority, and 5 was Essential. The survey was administered for the first time at the completion of session two. After completion of voting, the data was extracted from Jisc and analysed to determine the median of different innovations for determining ranking.

*Session three* began with a brief discussion, description and clarification of the list of innovations identified and their median ranking, demonstrating the overall consensus between participants. Afterwards, the same survey containing the list of innovations was administered for a second time, at the end of session three to observe any changes in overall prioritisation of innovations.

### Analysis

The transcripts of all sessions of the consensus meeting were read. An inductive and deductive thematic analysis approach [31] was used to analyse, identify, and categorise the innovations. The re-reading of the transcripts led the research team to familiarise themselves with the consensus of innovations, and a researcher (VHP) adopted an inductive coding using NVivo12. Induction uses “readings of raw data to derive concepts, themes, or a model through interpretations made from the raw data by an evaluator or researcher” [32]. The inductive thematic analysis enabled identification of list of innovations, recognised as themes. Later, deductive analysis was performed [33], which was informed by the attributes of innovations framework [34]. Table 2 summarises and categorises the broad themes of innovations emanating from the consensus workshop.

**Table 2:** Attributes of innovations

categories	Attributes of innovations	Innovations
Process	Roles	1. CFRs attending accident cases 2. CFRs attending falls patients (using riser) 3. CFRs’ social care and public welfare role 4. Retain the CFR role as it is now (no change) 5. Opportunities for different levels of contribution of CFRs 6. Differentiation and specialisation of CFR roles 7. Peer support/hot debrief 8. Counselling and support for CFRs 9. Distinction between types of CFRs (lay, fire etc.)
	Governance	10. National minimum standard (for governance etc.) 11. Reward and recognition 12. Clearer job description 13. Decentralisation in developing CFR policies 14. Insurance for the CFRs (older ages) 15. Better documentation and data for quality assessment 16. More funding support from ambulance service 17. Better communication from/with control room
	Training	18. Education of the community members by CFRs e.g. CPR 19. Ongoing training and mentorship 20. National volunteer certificate training 21. Standardised training 22. Portability of training 23. Specific mandatory and relevant training for CFRs
	Policies and protocols	24. Standard call sets 25. Support to leave at home 26. Dual CFR crew 27. Increase equality, diversity and inclusion in CFR schemes 28. Termination of resuscitation guidelines
	Recruitment	29. Increased number of volunteers
	Raising awareness	30. Creating awareness of CFRs through NHS 31. Creating awareness and promoting CFRs in the community
Technology	ICT	32. Navigation and communication gadget with live tracking 33. Radio with panic button 34. Handover e-form
	Transport	35. Dedicated car for CFRs 36. CFRs' access to blue light 37. Providing help with hospital transport
	Health technology	38. Entonox use 39. Blood glucose meter 40. Glucogel use

The ranking or prioritisation of innovations from the survey were exported to Microsoft Excel to analyse for consensus using composite score and descriptive statistics. The level of prioritisation was expressed as the median score and interquartile range for each innovation.

## Results

### Qualitative

In all, 26 members were present in the consensus meeting; 17 were participants, 11 of whom were representatives of seven English ambulance services, and six were PPI members. Nine researchers from the University of Lincoln facilitated the sessions.

Appendix 1 presents final reporting standard [35].Table 2 presents a comprehensive list of innovations identified from the qualitative analysis, and are presented in two broad categories: process innovations, and technological innovations (also see Appendix 1). Within the process innovations, we identified six attributes, and within the technological innovations, we identified three attributes.

#### Process innovations

Process innovations covered: roles; governance; training; policies and protocols; recruitment; and raising awareness. Examples of role innovations include: differentiation and specialisation of CFR roles. In terms of governance, participants suggested having rewards and recognition for CFRs. Participants also suggested training innovations, which included the standardisation and portability of CFR training. Increasing the number of volunteers and raising awareness were suggested by participants as innovations to improve recruitment.

For example, within broader process innovation, the innovation relating to the differentiation of specialisation of CFR roles was identified:

*“And I think in terms of the CFR role, we need to be going back to basics and defining what the role is going to do. It’s like you do one job and you do it well, rather than have somebody trying to do ten jobs and not do it well.”* (Ambulance 2, Group 2).

Similarly, distinction between types of CFRs was also recognised as a role innovation:

*“So, I was just echoing and absolutely agreeing that I think we need to split up the roles, rather than piling it all onto one single role, losing focus and making CFRs less effective in terms of what they were initially set up to do.”* (Ambulance 1, Group 2).

Reward and recognition for CFRs was recognised as governance innovation:

*“There should be mechanism of reward and public recognition of CFRs.”* (Ambulance 6, Group 2).

#### Technology innovations

Technological innovations included: ICT; transport; and health technology. The technological innovations were in relation to both software and hardware advancements, and these innovations were raised in the context of helping CFRs to respond faster to incidents and to simplify their role. Live tracking would help them get to incidents more effectively. Using Blue Lights would enable them to arrive on-scene faster. Being able to administer Entonox would increase their pain relief role.

For example, within broader technology innovation, the innovation relating to the digital handover form was identified:

*“That’s on a simple basis about how our volunteers can effectively gather information onsite and pass that back to remove clinicians so they can make choices they’re unable to make without being on scene because either the patients or the relatives don’t understand what questions needs to be asked or what information needs to be passed on.”* (Ambulance 6, Group 2).

Dedicated car for CFRs was an innovation in transport technology:

*“We’re also working through some trust-owned vehicles and having those available at set locations for the volunteers to be able to book and respond on.”* (CFR, Group 2).

Table 2 presents the comprehensive list of innovations we have identified.

The different innovations introduced and identified from the consensus workshop were discussed in the various sessions, and a diverse range of perspectives were brought into the discussions by the participants. Often, the varied perspectives of the participants were grounded in their professional background and experiences. For example, a dedicated car for CFRs as an innovation was introduced by the research team. This was identified in the empirical CFR study, and consensus participants agreed that having a dedicated CFR car was a high priority.

*“We’re also working through some trust-owned vehicles and having those available at set locations for the volunteers to be able to book and respond on… it could also mean they’d be able to move to areas or pockets of high demand outside of their communities that are under some additional pressure.”* (CFR, Group 2).

While participants agreed on the need for a dedicated CFR car, they recognised the challenges associated with this, especially within a rural context:

*“Having things like community responder vehicles—well, my group’s got a community responder vehicle, but if I want to use it, I’ve got to drive six miles to pick it up, and then someone’s got to pick it back up again. So, responder vehicles in rural settings aren’t always the answer, they’re really not. They’re fantastic in an urban area where responders are part of a tight-knight group, but in the rural community, they’re not really quite so relevant”* (PPI, Group 3).

The above excerpt suggests that although having a dedicated CFR car was considered vital for CFRs, the rural contextual factors present challenges for rural CFRs to access the resources. Similarly, the consensus participants disagreed about CFRs’ role in patient transport to a medical facility.

The CFR study participants emphasised the expansion of their role to include transporting patients from rural locations to the nearest medical facility. This innovation was considered important in circumstances when the ambulance is delayed due to the remote location, workload pressures for ambulance clinicians, the unavailability of ambulances and the rapid deterioration of patient conditions while they wait for an ambulance.

*“Taking patients to hospitals [by CFRs] is also a good idea for patients who are home and are critical, and waiting for ambulance for hours in rural areas.”* (PPI, Group 1).

The above excerpt demonstrates the expansion of CFR role was in line with the CFRs bridging the gap between the patients and EMS services while waiting for an ambulance in order to strengthen future rural health care. However, the consensus workshop participants perceived the innovation of CFRs transferring patients to the nearby medical facility as problematic, referring to the varying driving skills of CFRs and would not wish to see such a provision in the CFR schemes. For example, a consensus participant noted:

*“In terms of transport and double crewing… I’ve done it myself with SJ—but I just wouldn’t put that within the scope of the study.”* (Ambulance, Group 1).

Thus, it was apparent that the consensus stakeholders attributed different priorities to some of the innovations. Therefore, the ambiguities involved with recognising the significance of various innovations were identified, and the qualitative analysis has shown that recognising the significance of innovations was inherently subjective. As a result, the quantitative analysis was essential in determining the numerical indicators to recognise the importance of various innovations through voting and ranking, and it played a vital role in achieving the 3.2. Survey.

A total of 17 consensus participants took part in the first survey. The nine researchers facilitated discussions rather than taking an active part in the surveys. The results of the prioritisation revealed that: the counselling services; peer support for CFRs; requirement of communication gadgets with live tracking; standardised training; specific and mandatory training for CFRs; national minimum standards for governance; better communication with the control room; and increased number of volunteers, had a median of 4–5.

This suggests that, generally, respondents agreed that they were “essential priorities”. In contrast, the innovations, such as CFRs attending road accident cases, CFRs’ access to blue light, and patient transfer to hospitals were given the lowest priority. All of these innovations had a median of 1–2, which indicated an overall agreement of classifying these innovations as “not a priority”. See Appendix 1 for further information on how various innovations were prioritised in the first survey.

Group 1 met face-to-face with seven participants. It comprised of: two ambulance staff, a PPI member, and four researchers. Group 2 had nine online participants, which included four ambulance staff, two PPI members, and three in-person researchers. Group 3 also had nine online participants and comprised of four ambulance staff, three PPI members, and two in-person researchers. We ensured that all groups contained a mix of roles.

Sixteen consensus participants participated in the second survey. The results indicated that the most highly prioritised innovations were: communication gadgets with live tracking; better communication with the control room; creating awareness and promoting CFR in communities; standardised training; mandated and specific training for CFR; counselling support for CFRs; and peer support. While the lowest priorities were given to: CFRs’ access to blue light; and CFR roles in patient transfer to hospital by CFRs.

Appendix 1 shows that the round 1 and 2 scores were generally similar. The comparison of the round 1 and round 2 prioritisation highlighted that the following innovations increased in priority: standardised training; CFR roles in creating awareness and promoting CFR schemes in the communities; future requirement of specialisation in CFR roles; national volunteer certificate training; dedicated car for CFRs; and the future roles of CFRs attending falls patients using risers to specify a few. There were also innovations that decreased in priority, such as: more CFR recruitment; a requirement of electronic patient report form (EPRF); receiving more funding support from ambulance services; termination of resuscitation guidelines; CFRs having a social and public care role; and CFRs working as a dual crew.

### Divergence and convergence of qualitative and survey data

There were some innovations in the qualitative discussions that were ranked as one of the lowest priorities in the survey results and vice-versa. This demonstrates how the reflections of both qualitative themes and survey results provided convergent and divergent consensus among participants. As an example, an attribute identified in qualitative analysis highlighted that the use of Entonox by CFRs in pain management was a useful innovation. However, in both rounds of surveys it was ranked as a low priority (median 2—Appendix 1).

*“Like you say, in Y, it does work. Other areas don’t do it. Some people already give Entonox as part of their enhanced skills because CFRs want to do it. We’ve got some people that are really, really keen.”* (Ambulance, Group 1).

Similarly, transferring patients to hospital was considered a low priority (median of 1) in both rounds of the survey but was raised as a priority in the interviews.

*“Taking patients to hospitals [by CFRs] is also a good idea for patients who are home and are critical, and waiting for ambulance for hours in rural areas.”* (PPI, Group 1).

In contrast, counselling and support for CFRs were considered a significant innovation to support the mental health of CFRs, which may have been impaired by the traumatic experiences originating from the CFR roles. In both surveys, participants identified counselling and support for CFRs as an “important priority” (median 5—Appendix 1) and the qualitative analysis also indicates the significance of the innovations.

*“I think it’s really important that they get that mental health and wellbeing support. I think that’s important in any role, but particularly given what CFRs might end up seeing and having to deal with. I think their mental health is a top priority.”* (Ambulance, Group 3).

Similarly, better communication with the control room scored highly (median 5) in both rounds of the survey and was also considered important in the interviews. Thus, the consensus-building workshop identified and prioritised the set of innovations using a mixed-methods approach. The qualitative component explained innovations observed within the empirical CFR project and those recommended by the consensus participants. Concurrently, the quantitative approach facilitated the systematic ranking of these innovations, thereby providing insights into their prospective prioritisation for future CFR roles and governance.

## Discussion

### Summary of results

Using a four-stage mNGT, this study established consensus and ranked a set of innovations for improving the future provision of CFR schemes and future roles of CFRs for enhanced prehospital care and emergency services. In round 1, the following innovations were given the highest priority (median 5) by participants: peer support; counselling and support for CFRs; national minimum standards for governance; better communication with the control room; specific mandatory and relevant training for CFRs; increased number of volunteers; and communication gadget with live tracking. By contrast, the following innovations were given the lowest priority (median 1) in round 1: transferring patients to hospital; CFR access to blue light; and providing help with hospital transport.

In round 2, participants gave the following innovations the highest priority: peer support; counselling and support for CFRs; better communication with the control room; standardised training; specific mandatory and relevant training for CFRs; creating awareness and promoting CFRs in the community; and communication gadget with live tracking. By contrast, the participants gave the following innovations the lowest priority: transferring patients to hospital; and CFR access to blue light.

Between the survey and the qualitative results, there was divergence and convergence on a number of innovations. Using Entonox for pain management and transferring patients to hospital were considered important in the qualitative interviews but were not prioritised in the consensus workshop. Counselling and support for CFRs and better communication with the control room were considered important in both the interviews and the consensus workshop.

### Links to existing research evidence

Previous studies on CFRs focussed on the contribution of CFRs to emergency and prehospital care [10, 11], motivations of CFRs [5–7], role of CFRs in improving access to defibrillation [9]; reducing response times to out-of-hospital cardiac arrests [36], the demands on and stressors faced by CFRs [12], and the implementation of CFR schemes [4, 5]. This consensus study is the first of its kind to employ a mNGT [21, 22] to understand various innovations and rank them based on prioritisation of stakeholders and PPI.

Müller et al. study developed a reporting standard for smartphone-based dispatch of first responders [35]. While the localism of CFR schemes enables them to respond to local needs, there is limited reporting of how they operate. Our reporting standard presented in this study does not seek to establish minimum standards for how CFR schemes operate. However, it could be a step towards greater openness in reporting among CFR schemes and promote greater sharing of best practice between them.

### Implications for further research, policy, and practice

This consensus study informs guideline- and policy- stakeholders at national and regional ambulance service organisations about the significance of different innovations and how they might be used to improve CFR schemes throughout the country’s health services. Stakeholders who play crucial roles in implementing CFR schemes throughout the country recognised the innovations and their prioritisations, and were encouraged to implement change agendas in their respective organisations and regions. The study results, the list of innovations and their prioritisations are crucial for the health systems in guiding analogue CFR programmes and produce learning for future volunteerism in emergency and prehospital care. Previous studies identified problems in CFR schemes implementation, practices, and governance [5, 8, 10]. Our reporting standard, which represents a consensus among the key stakeholders with an interest in how CFR schemes operate, could promote greater transparency and facilitate greater co-operation in how they best serve their communities.

The results have important implications for future CFR policies and practices, which include aligning the roles of CFRs with their training and skills [6]. It also highlights the need for governance structures that provide adequate support to CFRs, including mechanisms for coordination, supervision, counselling, peer support, and communication.

The study emphasises the significance of recruitment strategies to attract and retain a diverse and skilled CFR workforce. Creating public awareness about the roles and capabilities of CFRs is crucial for improving community awareness, engagement, and support in the future [4]. The prioritisation of technological innovations for future CFR schemes to enhance communication and coordination among CFRs, control rooms, and other stakeholders can significantly improve timely and efficient care delivery [11]. Future studies should investigate whether and how these prioritised innovations are being implemented and establishing impacts.

This consensus-building study and the identified innovations can inform policy- and decision-makers on the future change agenda for CFR schemes. These results can influence policy development, resource allocation, and implementation strategies to improve CFR schemes and the quality of care delivered by CFRs in communities.

### Strengths and limitations

This is the first study to engage CFR stakeholders from across the country in co-creating different sets of innovations. It collectively recognises the innovations based on their significance and establishes  a consensus for strengthening future CFR roles.

This study contributed to the empirical understanding of innovations and provides the NHS, ambulance services, CFRs, patients, and public representatives with a common ground of knowledge on the current functioning and future potential of CFRs. The strength of this research lies in its utilisation of a highly specialised and varied panel, to co-create innovations based on the professional experiences, knowledge, and lived experiences of the panel members [37].

NGT methods have been criticised for their reliance upon the perceptions and individual professional experiences of the limited number of participants involved in the consensus [38]. While the diverse and expert participants in this study have methodological strengths, the subjective perceptions of the participants may have influenced their perceived recognition of importance of the innovations. However, participants with diverse professional expertise and lived experiences had equal anonymous opportunities to rank different innovations and contribute to prioritisation. Moreover, the triangulation of qualitative and quantitative data in this study provides both depth and breadth [39] to strengthen the reliability of the innovations and their prioritisations.

The patient and public voice, through the PPI representatives, accounted for only six of the 26 participants, which may suggest an under-representation. Future studies should seek to address the under-representation of PPI in consensus building initiatives in CFR schemes.

## Conclusion

This paper shows the convergence and divergence relating to the current roles of CFRs and current and future innovations for strengthening CFR schemes. The majority of innovation attributes showed at least one innovation designated as either “essential priority” or “high priority,” apart from the transport innovation. Within the attribute of transport innovation, which includes dedicated CFR cars, CFRs' access to blue light, and the provision of CFR support in hospital transport, these innovations were considered comparatively lower priorities. The specifics of these innovations demonstrate that, while all attributes of innovation were recognised as significant for future CFR provisions, those specifically associated with “counselling and support for CFRs,” “enhanced communication with the control room,” “improved communication and navigation devices,” “mandatory and standardised training,” and the establishment of national standards with improved awareness were broadly agreed to be high priority considerations for the future CFR provisions.

Prioritising some innovations over others within CFR schemes highlights the possibility of introducing new practices. This consensus paper contributes to developing a comprehensive list of innovations in CFR schemes, thereby informing future prospects on CFR roles. In addition, it emphasises the need for a set of innovations to address the unique challenges CFRs encountered in rural healthcare provision.

## Supplementary Information

Supplementary Material 1.

## Data Availability

The datasets generated and/or analysed during the current study are not publicly available due to the guidelines of the Research Ethics Committee, but are available from the corresponding author on reasonable request.

## Appendix 1: Joint display—prioritisation of the innovations

**Table:**

Innovations	#	Round 2	Round 1	Innovations excerpts derived from qualitative analysis
		Median [IQR]	Median [IQR]	
**Process**				
*Roles*				
CFRs attending accident cases	1	2 [1, 2.25]	2 [1, 3]	“The CFRs who already have worked with fire and police services are attending road traffic collisions.” (GP)
CFRs attending falls patients (using riser)	2	3 [2, 3.25]	2 [2, 3]	“Some CFRs were allowed to attend falls patients, and they were given riser chair, which is basically a lifting device to lift the fall patients.” (GP)
CFRs’ social care and public welfare role	3	2 [1, 3.25]	3 [2, 4]	“CFRs were engaged in social care work associated with health services such as delivering food and medicine, picking prescription for vulnerable population.” (GP)
Retain CFR role as it is now (no change)	4	3 [1, 4]	2 [1, 3]	“It was identified that there is an ongoing persistent concern about expanding the CFR role, and recommended that the CFR role should be maintained in its current form.” (GP)
Opportunities for different levels of contribution of CFRs	5	4 [2, 5]	4 [3, 4]	“I just want to reiterate what L put in the chat about the fact that, at her trust, they actually utilise CFRs to deploy CFRs because they know the CFRs’ skillset and can support them during deployment. So, that coincides with what IR’s saying as well.” (AB)
Differentiation and specialisation of CFR roles	6	4 [2, 5]	3 [3, 4]	“And I think in terms of the CFR role, we need to be going back to basics and defining what the role is going to do. It’s like you do one job and you do it well, rather than have somebody trying to do ten jobs and not do it well.” (BR)
Peer support/hot debriefs	7	5 [4, 5]	5 [4, 5]	“Well, the idea of a debrief has a very chequered history for one thing, and the other thing is that the consensus around debriefs after critical incidents and trauma is that the most important thing is actually the peer support that is available for emergency services. So, I think the emphasis should be on peer support, and that has the advantage—which is linked to the community—of being non-professional and it’s provided by others who know intimately through their own experience what it’s like to deal with these critical incidents and to live in certain communities, and so it de-professionalises it… Somebody mentioned community resilience, and those are the crucial words. You don’t go on a course and do community resilience; you get community resilience when you have cohesive communities.” (RO)
Counselling and support for CFRs	8	5 [5]	5 [4, 5]	“The need for counselling [is important]. We are lone people, and believe you and me, as a community responder, I’ve now dealt with three people that I knew who’ve died of a cardiac arrest. Yes, CFRs can be lonely, and I think that, in fact, it should be standard practice that after any CFR has dealt with a cardiac arrest where the outcome has been death, there should be an automatic phone call to that responder to help support them. Crews can support each other, CFRs can’t.” (MS)
Distinction between types of CFRs (lay, fire etc.)	9	4 [3, 4]	4 [3, 4]	“So, I was just echoing and absolutely agreeing that I think we need to split up the roles, rather than piling it all onto one single role, losing focus and making CFRs less effective in terms of what they were initially set up to do.” (VH)
*Governance*				
National minimum standards (for governance etc.)	10	4.5 [4, 5]	5 [4, 5]	“There should be national standardisation so people can move around, and so the public knows that when a CFR attends, they’ve had some sort of training. But the roles should be defined, because as MS said, there are a lot of CFRs that do only want to operate in their own community, and there are some that are looking at it as a progression to another career. So, there should be defined roles, but there should be national standardisation. And the same goes for the way episodes are coded. One of the problems that arises when it comes to auditing and doing research is that you need to have standardisation.” (AB)
Reward and recognition	11	4 [2.75, 5]	4 [2, 5]	“There should be mechanism of reward and public recognition of CFRs.” (NM)
Clearer job/role description	12	4 [3, 5]	4 [3, 5]	“There becomes a point where we actually need to go back to basics and ask ourselves what the role is about and what we’re wanting them to achieve. Do we want our CFRs to go to category 1 calls? Do we want them to go to category 3 and category 4 calls? Do we want them to go out and man welfare stations? What is it that we’re wanting from our 4 or 5 h a week?” (BR)
Decentralisation in developing CFR policies	13	2 [1, 3]	2 [1, 3]	“Especially CFR have noted that they should be included in the development of CFR guidelines in their respective Ambulance services. They have called for a more decentralisation in devising policies, including training policies, and the CFRs should be included in decisions making processes on policies or guidelines which directly affect their work.” (GP)
Insurance for the CFRs (older ages)	14	4 [2.75, 5]	4 [3, 5]	“To get back to the age thing, those of us who are over 70 are not insured. NHS personal accident insurance stops at 70. So, as a responder, if I get injured, I’m not insured. I think this is totally unfair, I really do” (MS)
Better documentation and data for quality assessment	15	4 [3, 5]	4 [4, 5]	“Also as far as recording [documentation] things and making sure that in the future, when we want to make sure things are working properly, we can audit them properly.” (AB)
More funding support from ambulance service	16	3 [3, 4.25]	4 [3, 4]	“Funding has been one of the major area of exploration in our data. It is perceived and experienced that there should be more funding support from the ambulance services, so the CFRs would be less reliant on fundraising and Charity.” (GP)
Better communication from/with control room	17	5 [4, 5]	5 [4, 5]	“In terms of the communication between the control room as well and the CFRs, it was found that there should be better communication about the patients’ conditions, Signs and symptoms from the control room to the CFRs. This would minimise the mismatch in patient information received from control room and what CFRs observe on scene.” (GP)
*Training*				
Education of community members by CFRs e.g. CPR	18	4 [3, 5]	4 [3, 5]	“For me, that’s the core bit we’ve got to get right. How do we get more people in their community available to do CPR, available to go to that patient when they’re having a cardiac arrest? That’s the most fundamental thing we can do.” (NH)
Ongoing training and mentorship	19	4 [3, 5]	4 [4, 5]	“They may be able to dual crew, which could also provide mentoring opportunities.” (VH)
National volunteer certificate training	20	3.5 [2, 4]	3 [2, 4]	“When you move an internal certificate to another organisation, it doesn’t mean anything. Actually, that portability level helps with some of our recruitment and retention because some of our volunteers might suddenly want to join IR in the Isle of Wight and go and see some sunshine. But actually, can they move their volunteering experiences to other organisations?” (LH)
Standardised training	21	5 [4, 5]	4 [4, 5]	“They’ve had some sort of training… but there should be national standardisation.” (AB)
Portability of training	22	3.5 [2.75, 4]	3 [2, 4]	“In terms of portability within services, in-house training courses mean nothing to anyone else. When you move an internal certificate to another organisation, it doesn’t mean anything. Actually, that portability level helps with some of our recruitment and retention because some of our volunteers might suddenly want to join IR in the Isle of Wight and go and see some sunshine.” (LH)
Specific mandatory and relevant training for CFRs	23	5 [4, 5]	5 [4, 5]	“As a responder, I’m being given training in child abuse. I don’t think that giving me an hour’s course on child abuse gives me the right to make those kinds of judgements. So, I think we need to make sure that training is very, very relevant. To give a CFR fire training on evacuating patients from hospital is totally irrelevant, but I had to do it to stay as a CFR. So, we really do need to consider that.” (MS)
*Policies and protocols*				
Transferring patients to hospital		1 [1, 2]	1 [1, 2]	“The CFRs should be able to transfer the patients to nearby hospital particularly in remote areas where ambulance arrival time is more than 30 min. And that would also reduce the cost of patient transfer as compared to allocation of an ambulance.” (GP)
Standard call sets	24	4 [2.75, 5]	4 [3, 5]	“So, within the West Midlands… CFRs have been able to book on to respond to either their full call set or just category one calls, i.e. when they’re booked on, they’ll respond to category 1 calls and will leave this meeting if there’s a category one call around the corner from them. We have that as an option or at least we certainly did have that as an option for CFRs.” (JM)
Support to leave at home	25	3.5 [1, 4]	3 [2, 4]	“The support would be in terms of acuity. That’s what we’re working on with our low-acuity trial at the moment. So, we send a CFR to somebody who may have pressed an alarm or who has fallen but doesn’t require hospital. They then get triaged by the clinician, so the CFR wouldn’t make that decision.” (M)
Dual CFR crew	26	1.5 [1, 3]	2 [1, 3]	“They may be able to dual crew, which could also provide mentoring opportunities.” (VH)
Increase equality, diversity and inclusion in CFR schemes	27	4.5 [2.75, 5]	4 [2, 5]	“As we saw for some reason majority of white population are receiving the benefits of CFR care. In future that needs to go beyond that boundary.” (AB)
Termination of resuscitation guidelines	28	2.5 [1.75, 4]	3 [3, 4]	“Since the CFRs cannot declare a patient dead, they have to perform CPR on patients who are already dead. So, the innovation for change was to terminate the CFR resuscitation guideline.” (GP)
*Recruitment*				
Increased numbers of volunteers	29	4 [4, 5]	5 [4, 5]	“I think we need to grow that pool of volunteers first… So, the more people we have available to do that, the better outcomes we’ve got for patients having out-of-hospital cardiac arrests.” (NH)
*Raising awareness*				
Creating awareness of CFRs through NHS	30	4 [3, 4]	4 [3, 4]	“Another area of change emerged is that there is a requirement of more awareness in the rural areas about the CFR schemes. And, one-of-a-kind way suggested by the participants is that the GP surgery should be involved in creating awareness about CFR schemes. So when the patients or the relatives visit the GP surgeries, they would be informed that there are availabilities of CFRs in their areas and the common roles and responsibilities. Similarly, in order to create more awareness, there should be distribution of leaflets in the Community by CFRs.” (GP)
Creating awareness and promoting CFRs in community	31	5 [4, 5]	4 [4, 5]	“You need to be reaching out to the community… There’s a lot of willingness, I think, in the community to help out, but somebody has to be set up to actually help us as community members get involved, get trained and start doing things that are useful.” (NF)
**Technology**				
*ICT*				
Navigation and communication gadget with live tracking	32	5 [4, 5]	5 [4, 5]	“A contemporary navigation map helps CFRs to find the location of patients easily, especially in remote and rural areas. The navigation map highlights the name of the house, as well as automatically navigate the CFRs to reach the patients.” (GP)
Radio with panic button	33	4 [3, 5]	4 [3, 5]	“A radio with a panic button, is a significant innovation for ensuring safety of the CFRs, which would be used in hostile situation. For example, there was an incident where a CFR was held hostile and in that situation the CFR tried to call the control room asking for help but the call was missed. So, having a radio with a panic button will help to improve the security of the CFRs in such situations.” (GP)
Handover e-form	34	3.5 [3, 5]	4 [2, 5]	“That’s on a simple basis about how our volunteers can effectively gather information onsite and pass that back to remove clinicians so they can make choices they’re unable to make without being on scene because either the patients or the relatives don’t understand what questions needs to be asked or what information needs to be passed on.” (NM)
*Transport*				
Dedicated car for CFRs	35	3 [2, 4.25]	2 [2, 3]	“Dedicated CFR cars were identified as another important attribute in the functions of CFRs for their ease at travelling and reaching out to patients.” (GP)
CFR access to blue light	36	1 [1, 1.5]	1 [1, 2]	“Findings suggests that the CFRs should be able to use blue light, which will enable the CFRs to reach at the patients early, while getting around traffic and delays. On the other hand, some suggested Blue light access for all CFRs might pose risk for the CFR themselves as well as others on road since it requires a high level of driving skills.” (GP)
Providing help with hospital transport	37	2 [1, 3]	1 [1, 2]	“The CFRs should be able to transfer the patients to nearby hospital particularly in remote areas where ambulance arrival time is more than 30 min. And that would also reduce the cost of patient transfer as compared to allocation of an ambulance.” (GP)
*Health technology*				
Entonox use	38	2 [1, 4]	2 [2, 3]	“The Entonox as a painkiller for the CFRs to use in cases of emergencies has appeared as another important innovation.” (GP)
Blood glucose meter	39	4 [3, 5]	4 [3, 5]	“Similarly, in the area of equipment, some participants have stated that they should be able to access diabetes or blood sugar level test kits. At the same time, they should be able to get glucogel medications for patients with low blood glucose levels for timely and appropriate care.” (GP)
Glucogel use	40	4 [2, 4.5]	4 [2, 4]	“Similarly, in the area of equipment, some participants have stated that they should be able to access diabetes or blood sugar level test kits. At the same time, they should be able to get glucogel medications for patients with low blood glucose levels for timely and appropriate care.” (GP)

## Abbreviations

BLS: Basic life support
CFR: Community first responder
EMS: Emergency medical service
EPRF: Electronic patient report form
mNGT: Modified nominal group technique
MS: Microsoft
NGT: Nominal group technique
NHS: National Health Service
PPI: Patient and public involvement
